# Supplementary figures and images for: A graph theory approach to analyze birth defect associations
Source: PLoS One. 2020 May 22;15(5):e0233529. doi: 10.1371/journal.pone.0233529 (PMC7244144; doi:10.1371/journal.pone.0233529)

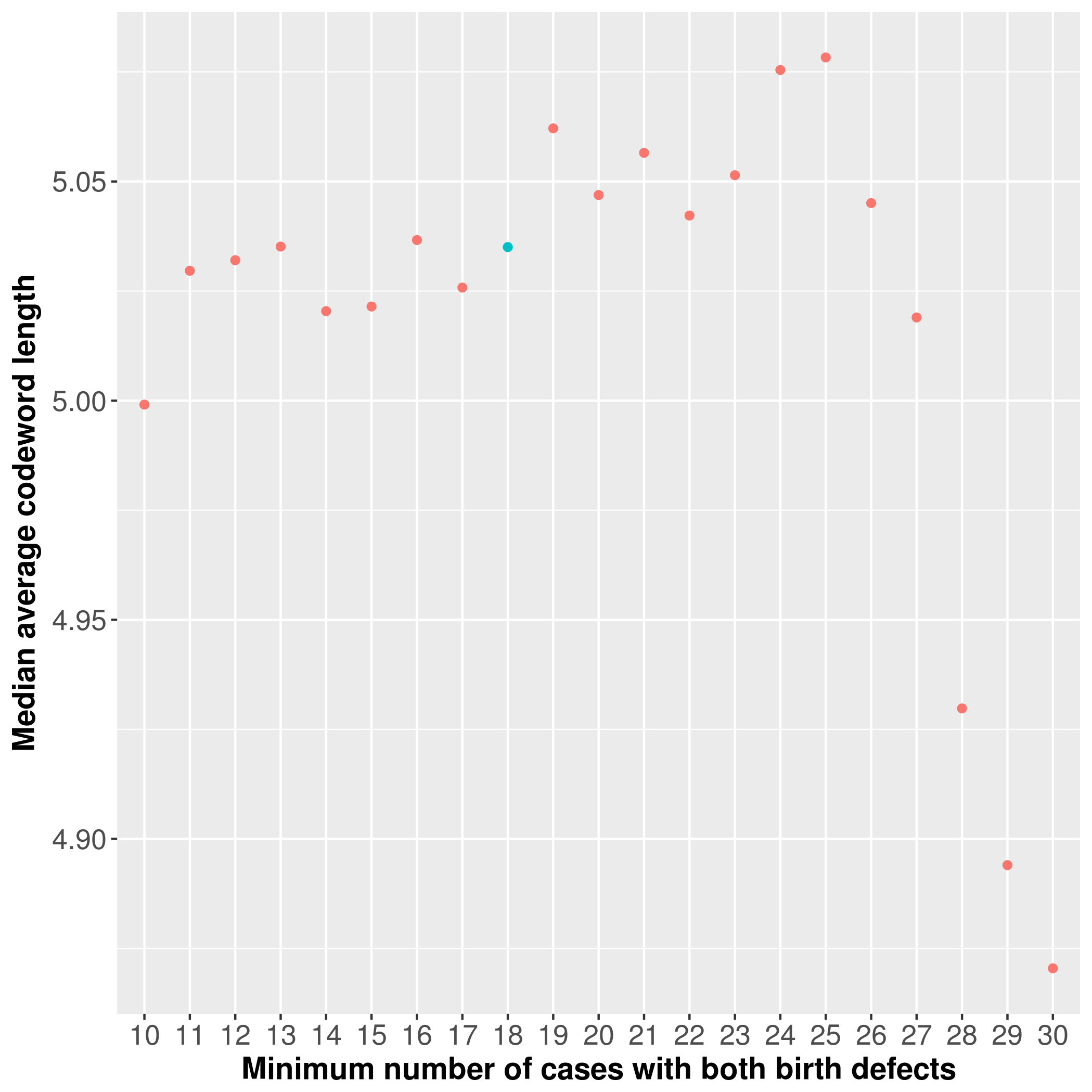

Supplement: S1 Fig — The median average codeword length corresponds to the partition of weighted graphs generated with the VA-Chi2 function and different threshold values: I) minimum number of cases with both defects between 10 and 30, with a step of 1; A) number of edges (with greater strength of association) included in the graph, between 50 and 800 with a step of 25. The blue dot was the threshold selected in this work. (TIF) [file pone.0233529.s001.tif]

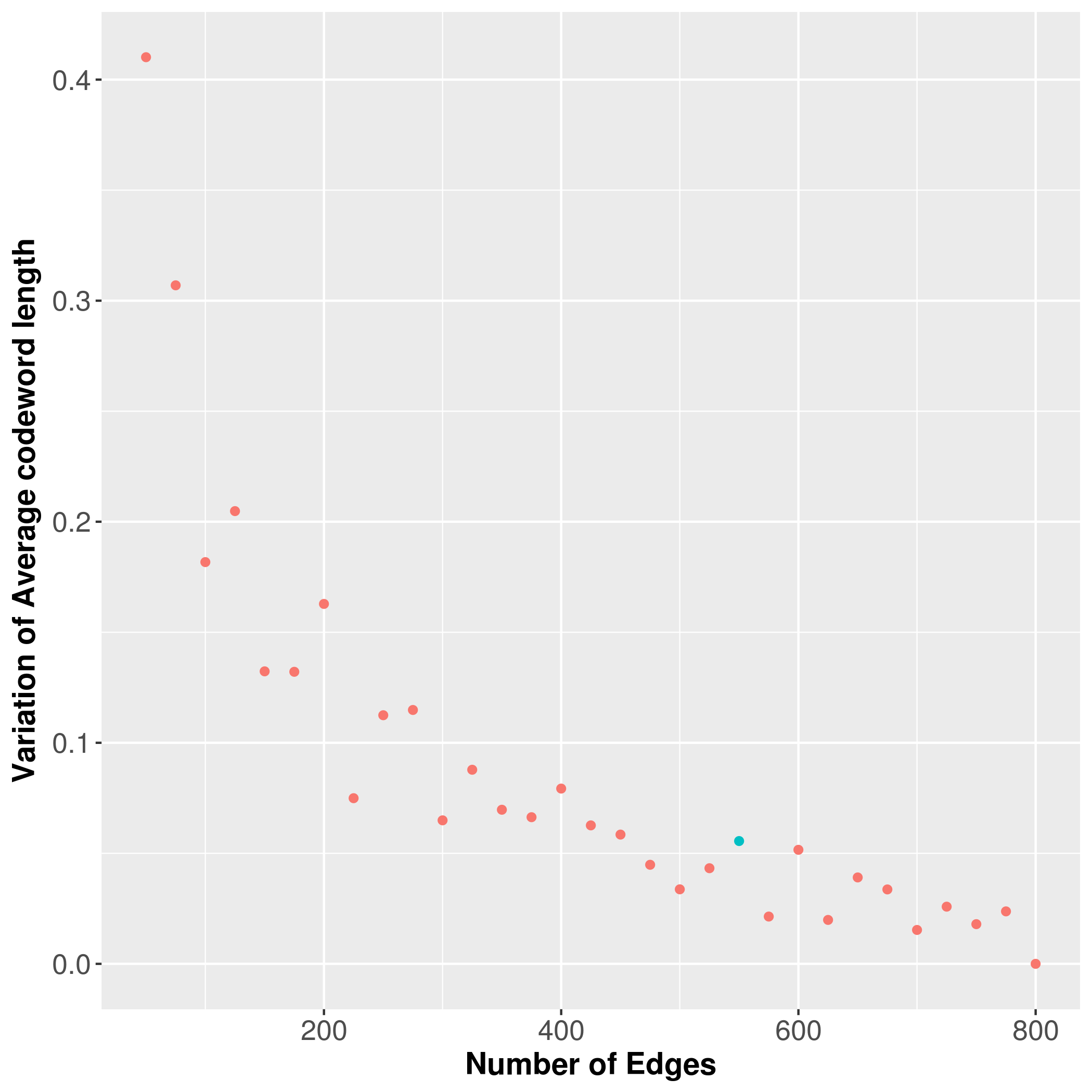

Supplement: S2 Fig — The average codeword length corresponds to the partition of weighted graphs generated with the VA-Chi2 function, a minimum number of cases with both defects of 18, and number of edges (A) (with greater strength of association) included in the graph, between 50 and 800 with a step of 25. The variation of ACL for each value of A was calculated with respect to the previous value of A (ordered from highest to lowest). A positive variation indicates a decrease in the ACL. The blue dot was the threshold selected in this work. (TIF) [file pone.0233529.s002.tif]
