# Supplementary material for: A graph theory approach to analyze birth defect associations
Source: PLoS One. 2020 May 22;15(5):e0233529. doi: 10.1371/journal.pone.0233529 (PMC7244144; doi:10.1371/journal.pone.0233529)
Supplement: S1 Appendix — (DOCX) [file pone.0233529.s008.docx]

**S1 Appendix. Methodological details.**

**Association functions**

To determine the association strength between two birth defects (BD), the following functions were evaluated: Dice, Likelihood Ratio test, Pointwise Mutual Information, Minimum Sensitivity and Chi-Squared [1,2]. This last one, which has been applied in previous studies [3], was chosen because it showed the best graph partition quality (Figure 1). For all functions, the total number of cases was used as denominator. Only associations that occurred more frequently than expected by chance were included under the assumption of independence.


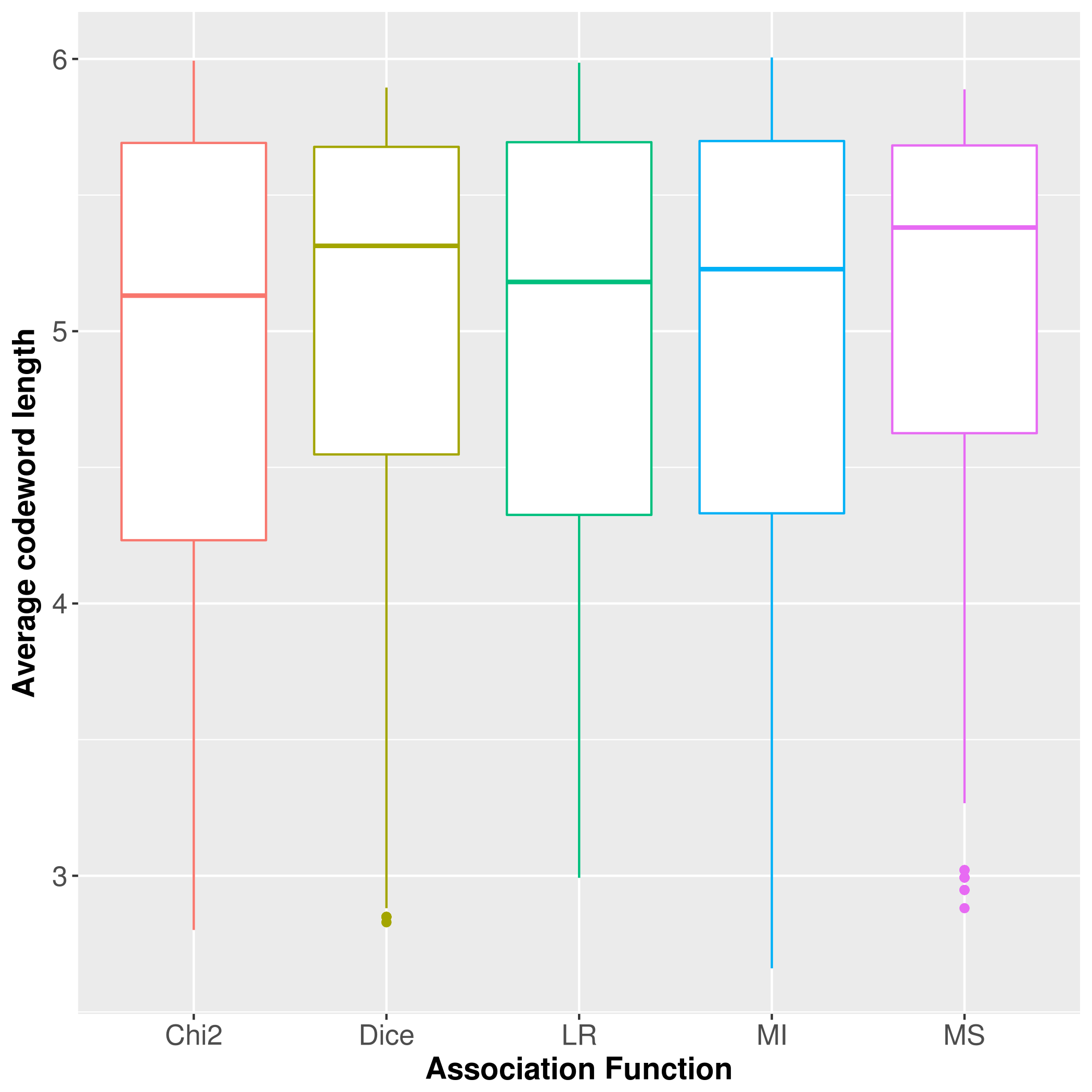


**Figure 1.** Boxplot of average codeword length for each association function. For each function, unweighted graphs with different threshold values were generated: I) minimum number of cases with both defects between 10 and 30, with a step of 1; A) number of edges (with greater strength of association) included in the graph, between 50 and 800 with a step of 25. Then each graph was partitioned with Infomap and the average codeword length was determined. Chi2: Chi-Squared independence test, Dice: Dice distance, LR: likelihood-ratio test, MI: Pointwise Mutual Information and MS: Minimum Sensitivity.

**Reference graph models**

To create graphs with the Erdos & Renyi (1959) [4], Barabási & Albert (1999) models [5], and random graphs with the same degree and weight distributions, the functions from the igraph R package (version 1.2.2), were used [6].

**Degree distribution**

The degree distribution was evaluated using the methodology suggested by Clauset et al. (2009), (poweRlaw R package) which estimates the goodness-of-fit based on the Kolmogorov-Smirnov statistics and the significance of the adjustment with the bootstrapping procedure using 10,000 simulations [7].

**References**

1. Kolesnikova O. Survey of word co-occurrence measures for collocation detection. Comp. y Sist. 2016;20(3): 327-344. doi: dx.doi.org/10.13053/cys-20-3-2456

2. Cao H, Hripcsak G, Markatou M. A statistical methodology for analyzing co-occurrence data from a large sample. J. Biomed. Inform. 2007;40(3): 343-52. doi.org/10.1016/j.jbi.2006.11.003

3. Zhou X, Menche J, Barabási AL, Sharma A. Human symptoms-disease network. Nat Commun. 2014; 5: 4212. doi:10.1038/ncomms5212

4. Erdos P, Rényi A. On Random Graphs I. Publicationes Mathematicae (Debrecen). 1959;6: 290-7.

5. Barabási AL, Albert R. Emergence of scaling in random networks. Science. 1999;286(5439): 509-512. doi: 10.1126/science.286.5439.509

6. Csárdi G, Nepusz T. The igraph software package for complex network research. Inter. J. Comp. Syst. 2006;1695(5): 1-9.

7. Clauset A, Shalizi CR, Newman ME. Power-law distributions in empirical data. SIAM Review. 2009;51(4): 661-703. doi: [10.1137/070710111](https://arxiv.org/ct?url=https%3A%2F%2Fdx.doi.org%2F10.1137%2F070710111&v=b4b21105)
