# Supplementary material for: A graph theory approach to analyze birth defect associations
Source: PLoS One. 2020 May 22;15(5):e0233529. doi: 10.1371/journal.pone.0233529 (PMC7244144; doi:10.1371/journal.pone.0233529)
Supplement: Attachment — Submitted filename: Response to Reviewers.docx [file pone.0233529.s009.docx]

*3. Have the authors made all data underlying the findings in their manuscript fully available?*

*The PLOS Data policy requires authors to make all data underlying the findings described in*

*their manuscript fully available without restriction, with rare exception (please refer to the*

*Data Availability Statement in the manuscript PDF file). The data should be provided as part*

*of the manuscript or its supporting information, or deposited to a public repository. For*

*example, in addition to summary statistics, the data points behind means, medians and*

*variance measures should be available. If there are restrictions on publicly sharing data—*

*e.g. participant privacy or use of data from a third party—those must be specified.*

*Reviewer #1: No*

**Reply**: Data supporting manuscript results are summarized in the supplemental tables

(description of birth defects codes, edges included in the Birth Defects Graph, nodes of each

partition group and their centrality indices) and figures (boxplot of average codeword length

for each association function, median average codeword length by minimum number of

cases with two birth defects, variation of average codeword length by number of edges).

ECLAMC individual registries cannot be included due to institutional data sharing

restrictions.

*4. Is the manuscript presented in an intelligible fashion and written in standard English?*

*PLOS ONE does not copyedit accepted manuscripts, so the language in submitted articles*

*must be clear, correct, and unambiguous. Any typographical or grammatical errors should be*

*corrected at revision, so please note any specific errors here.*

*Reviewer #1: No*

**Reply**: The new version of the manuscript has been revised by our institutional proofreading

and editing service.

*5. Review Comments to the Author*

*Please use the space provided to explain your answers to the questions above. You may*

*also include additional comments for the author, including concerns about dual publication,*

*research ethics, or publication ethics. (Please upload your review as an attachment if it*

*exceeds 20,000 characters)*

*Reviewer #1: In this manuscript the authors apply graph theoretic methods to the analysis of*

*patterns of birth defects diagnoses in a large collaborative birth defects surveillance*

*database. The paper holds some interest, but should be framed in a broader context and*

*substantially revised. Some specific comments appear below.*

*First, the title and throughout refers to 'The Graph Theory'. Actually what the authors do is to*

*apply graph-theoretic methods to analysis of birth defects diagnoses. There are other*

*approaches under exploration that take a similar approach, using machine learning methods.*

*The authors should discuss their approach within this context, and describe how it differs*

*and what advantages if any it offers. All of these approaches are agnostic in that they don't*

*take into account things we might know about diagnoses, so the value-add from the*

*approach outlined here should be interpreted in context as well.*

**Reply:** The aim of this work was not to compare different methods but to establish the

usefulness of the Graph Theory for the analysis of birth defects associations in a large-scale

database. Previous research papers describing this approach have been published, they

have however not focused on birth defects. Their references are included in the manuscript.

We thank the reviewer for this comment, which was of importance for redesigning the

manuscript.

*Second, throughout the text there are poorly constructed sentences, mis-spelling,*

*statements that are not as well referenced as they might be. Taking just the abstract and first*

*page of the body of the manuscript as examples:*

*In the abstract, all acronyms should be spelled out, and the actual acronym used only if*

*referenced more than once. Saying the findings 'seems to validate' the proposed method is a*

*bit weak.*

**Reply:** The abstract has been revised and corrected.

*On p 3, line 54, one of these references is from 1983?*

**Reply:** Yes, it is correct.

*line 55 - what is meant by thalidomide and rubella 'episodes'? Thalidomide has been*

*strongly linked with the development of birth defects surveillance, and there is a literature to*

*support this (none cited), but this is less true for congenital exposure to Rubella, which came*

*to attention decades before any substantial activity in birth defects surveillance ensued.*

**Reply:** Rubella has been eliminated from the text.

*line 59, would read better as 'Most studies have used one of two approaches to analyze*

*associations between unrelated birth defects.' Then, 'One approach focuses . . .'*

**Reply:** We have changed the text, following the suggestion

*lines 60-62, this sentence seems to over-generalize*

**Reply:** The sentence was rearranged.

*line 62, you mean 'multivariable' rather than 'multivariate'*

**Reply:** The answer is No. The expression “multivariate methods” has been extracted from

the research paper cited in the manuscript.

*line 73, should use 'have' rather than 'has' to refer to 'vast amount'*

**Reply:** It has been corrected.

*The above comments refer to a single page in the manuscript. This reviewer could make*

*similar recommendations throughout, and suggests that the entire manuscript be copyedited*

*by a scientist familiar with birth defects surveillance who has English as a first language.*

**Reply:** The text has been revised

*Additional comments:*

*p 4 line 91-94: it doesn't necessarily follow that, if the graph-theoretic approach works well to*

*identify known associations it will therefore be able to identity novel associations.*

**Reply:** It does not necessarily follow but it can be expected. The sentence has been

modified

*p 5, line 103 - so the available data is continually coded in ICD8, even to the present?*

**Reply:** Yes. ICD8 has always been used, in order to maintain homogeneity in birth defects

codifications over time

*line 112, 117 and elsewhere. What is the volume-adjusted Chi-square test? A little more*

*detail would be helpful to readers.*

**Reply:** A more detailed volume-adjusted Chi-square test description was included in the

revised version of the manuscript.

*line 123 what about associations requiring more than two birth defects - how does this*

*method aggregate?*

**Reply**: The method starts with the minimum number (i.e. 2) of associated defects. More than

two are studied through their decomposition into pairs.

*The section on methods contains a great deal of detail, more than is needed in the body of*

*the manuscript. Consider placing some of this material in an appendix, and referring only to*

*the terms and methods needed to describe the results in the methods section.*

**Reply:** We have followed the suggestion and some of the material in the Methods Section

has been placed as Supplemental Material.

*Table 1, p 8-9, the first two rows are repetitive across all columns. Place in the title or in a*

*note at bottom of table instead.*

**Reply:** Table 1 has been corrected.

*p 9 - lots of VA-Chi2 values in text. What's required for one to be significant? 3.84??*

**Reply**: The associations included in the graph were not determined by the VA-Chi2

significance but by the variation in the partition quality. Therefore, a threshold of significance

for VA-Chi2 was not established.

*Some of the material in the discussion belongs in results, for example lines 270-272 on p 11.*

*The discussion is not the place to introduce findings not previously mentioned elsewhere.*

**Reply:** We have moved the referred text to the Results section

*This reviewer did not comment in detail on the discussion. But its hard to know whether*

*these results 'seem to validate' the approach (p 14 line 336). To do this, it would be better to*

*directly compare this method with other methods such as machine learning, discriminant or*

*clustering analyses, etc, and show that it is similar or superior to those approaches.*

**Reply:** At this point, we disagree. Comparison would show which method is better, while

validation means to establish if the method is able to identify associations. As stated at the

beginning, it was beyond the aim of this work to compare the graph theory with other

approaches.
